# Choosing the Right Extracellular Vesicle: Cross-Kingdom Immunological Functions Linking Molecular Mechanisms to Therapeutic Applications

**DOI:** 10.3390/biom16060919

**Published:** 2026-06-20

**Authors:** Boglárka Schilling-Tóth, Daiana Alymbaeva, Krisztián Németh, Dávid Sándor Kiss, István Tóth, Gábor Andócs, Ondrašovičová Silvia, Brigitta Tagscherer-Micska, Gergely Jócsák, Tibor Bartha

**Affiliations:** 1 Department of Physiology and Biochemistry, University of Veterinary Medicine Budapest, István u. 2., H-1078 Budapest, Hungary; schilling-toth.boglarka.maria@univet.hu (B.S.-T.); daiana.alymbaeva@univet.hu (D.A.); nemeth.krisztian@univet.hu (K.N.); kiss.david@univet.hu (D.S.K.); toth.istvan@univet.hu (I.T.); bartha.tibor@univet.hu (T.B.); 2 Department of Microbiology and Infectious Diseases, University of Veterinary Medicine Budapest, Hungária krt. 23-25., H-1143 Budapest, Hungary; andocs.gabor@univet.hu; 3 Department of Biology and Physiology, University of Veterinary Medicine and Pharmacy in Košice, Komenského 73, 04181 Košice, Slovakia; silvia.ondrasovicova@uvlf.sk; 4 Department of Immunology, Institute of Biology, Eötvös Lóránd University, Pázmány Péter sétány 1/a, H-1117 Budapest, Hungary; brigitta.micska@ttk.elte.hu; 5 National Laboratory of Infectious Animal Diseases, Antimicrobial Resistance, Veterinary Public Health and Food Chain Safety, University of Veterinary Medicine, H-1078 Budapest, Hungary

**Keywords:** mammalian extracellular vesicle, bacterial extracellular vesicle, plant extracellular vesicle, immunological response, EV therapy, vesicle generation, adjuvant, immune regulation

## Abstract

Extracellular vesicles (EVs) are key mediators of intercellular communication across biological kingdoms, with central roles in immune regulation and disease processes. Despite shared structural features, EVs derived from bacteria, plants, and mammalian cells differ substantially in their biogenesis, molecular composition, and immunological functions. EV formation pathways generate vesicles with distinct cargo profiles, including pathogen-associated molecular patterns (PAMPs) in bacterial EVs, regulatory small RNAs in plant-derived vesicles, and cytokines, microRNAs, and antigen-presenting complexes in mammalian EVs. Differences in cargo result in divergent immune outcomes. Bacterial EVs predominantly activate innate immunity via pattern recognition receptors such as Toll-like receptors, whereas plant-derived EVs exhibit low immunogenicity and mediate cross-kingdom RNA interference. In contrast, mammalian EVs primarily regulate immune responses by modulating antigen presentation and cytokine signaling. These findings support a framework in which EV origin determines immunological function and therapeutic applicability. This perspective highlights the importance of selecting appropriate EV sources for vaccine development, regenerative medicine, and targeted delivery strategies, while addressing current challenges related to heterogeneity, standardization, and safety.

## 1. Introduction

Extracellular vesicles (EVs) are membrane-bound nanoparticles released by nearly all cell types across different biological kingdoms and are increasingly recognized as essential mediators of cell-to-cell signaling. Once thought to be cellular debris, EVs are now understood to be involved in a wide array of physiological and pathological activities, including immune regulation, tissue maintenance, microbial interaction, and host–pathogen dynamics [1,2,3,4,5].

The remarkable heterogeneity of EV populations has led to ongoing challenges in classification and nomenclature [1,6]. According to the latest MISEV23 guidelines, the nomenclature for vesicles has been refined to promote clarity and precision in the field. The term ‘extracellular vesicles’ (EVs) is defined as particles enclosed by a lipid bilayer that are incapable of autonomous replication, representing the vesicular component of extracellular particles [1]. While operational terms are recommended for describing vesicles, researchers are advised to use these terms cautiously, as separation methods may influence their accuracy. Biogenesis-related terminology should be avoided unless the specific subcellular origin of an extracellular vesicle can be demonstrated, given that most research addresses a broad population of EVs rather than distinct subtypes such as ectosomes or exosomes. In this context, ‘extracellular particles’ is used as an overarching term for cell-derived multimolecular assemblies ranging from nanometer to micron scale, encompassing both vesicular and non-vesicular entities, while ‘non-vesicular extracellular particles’ specifically refers to the non-vesicular fraction. These definitions are based on structural characteristics, independent of source or intended application, and reflect the most current recommendations for the accurate classification of vesicles [1].

Vesicles differ substantially in size, membrane composition, molecular cargo, and mechanisms of biogenesis, while considerable overlap exists between traditionally defined EV subpopulations [1,7]. Historically, EVs have often been categorized according to size and presumed origin, resulting in classifications such as exosomes, microvesicles, and apoptotic bodies [6]. However, accumulating evidence indicates that size alone cannot adequately predict EV function [1]. Although EV size influences physical characteristics such as cargo capacity and cellular uptake, increasing evidence indicates that cargo composition and biological function are primarily determined by biogenesis and the physiological state of the parent cell. For example, large mammalian EVs may contain mitochondrial components, cytokines, or damage-associated molecular patterns (DAMPs), whereas bacterial vesicles of comparable size can be enriched in pathogen-associated molecular patterns (PAMPs) and virulence factors, and plant-derived EVs (PDEVs) may carry regulatory small RNAs, defense-related molecules, and bioactive metabolites. Consequently, EV function emerges from the combined influence of size, cargo composition, membrane architecture, and biological origin rather than any single characteristic alone [5,6,7].

Interestingly, many of the therapeutic applications currently being explored for EVs appear to reflect these native biological functions. Mammalian EVs are primarily investigated for regenerative and immunomodulatory therapies, bacterial extracellular vesicles—outer membrane vesicles (OMVs)—have emerged as attractive platforms for vaccines and immune stimulation, whereas PDEVs are increasingly studied as oral delivery vehicles for bioactive compounds and RNA-based therapeutics [8,9,10,11]. Although these relationships are not necessarily causal, they suggest that the physiological functions fulfilled by EVs in their source organisms may provide valuable insight into their translational strengths and limitations.

Despite rapidly growing interest in EV biology, the relationship between EV origin, physiological function, and therapeutic applicability remains incompletely understood. Although EVs from mammalian, bacterial, and plant systems share common features as membrane-bound carriers of biological information, they arise through distinct biogenetic pathways, carry different molecular cargoes, and fulfill different physiological functions within their source organisms. These differences are increasingly reflected in their emerging biomedical applications, ranging from regenerative and immunomodulatory therapies to vaccine development and targeted delivery strategies. Therefore, this review adopts a cross-kingdom perspective to examine how EV origin shapes vesicle composition, immune interactions, and translational applicability, with particular emphasis on mammalian recipient systems. By integrating these aspects within a comparative framework, the review aims to provide a broader understanding of the relationship between biological origin, physiological function, and therapeutic potential.

## 2. Size and Origin-Specific Extracellular Vesicle Generation

**Figure 1:**
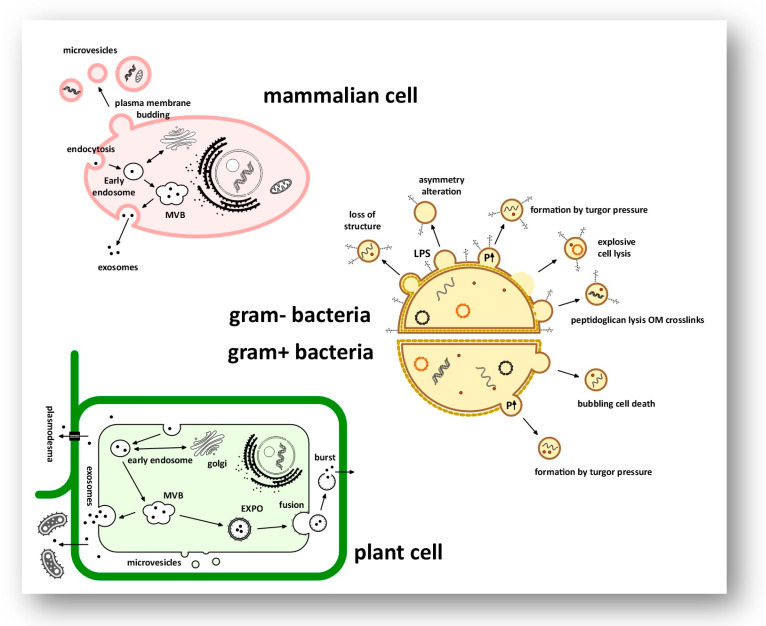
Schematic overview of extracellular vesicle (EV) biogenesis in mammalian, bacterial, and plant cells.

Although membrane-based long-distance communication via EVs is not exclusive to mammalian organisms, it is also well established in plants, while the most extensive literature is available for humans and other mammals [12] and for bacterial cells [13] (Figure 1). The following section discusses EV generation across biological kingdoms.

### 2.1. Mammalian Extracellular Vesicles (MEVs)

In mammalian systems, EVs comprise a heterogeneous population of membrane-bound structures that differ in biogenesis, size, and functional properties [14]. In this section, the classification of mammalian EVs is discussed with emphasis on size and biogenetic origin, as these features are closely linked to vesicle composition and function. The major EV subtypes include exosomes, microvesicles, and apoptotic bodies, each generated through distinct cellular pathways and characterized by specific size ranges and molecular compositions [14].

Exosomes are small vesicles, typically 30–150 nm in diameter, that are generated through the endosomal pathway [15]. Their formation begins with the inward budding of the limiting membrane of early endosomes, leading to the generation of intraluminal vesicles within MVBs [16]. This process is regulated by both Endosomal Sorting Complexes Required for Transport (ESCRT)-dependent mechanisms, involving proteins such as Tumor Susceptibility Gene 101 (TSG101) and Programmed Cell Death 6-Interacting Protein (ALIX), and ESCRT-independent pathways, including ceramide-driven membrane curvature [17,18,19]. When MVBs fuse with the plasma membrane, the intraluminal vesicles are released into the extracellular space as exosomes, a step regulated by RAB proteins [20]. Their cargo, including proteins, lipids, and nucleic acids, is selectively sorted and reflects the origin and physiological state of the parent cell [21].

In contrast, microvesicles, also referred to as ectosomes, are larger vesicles, typically ranging from approximately 100 nm to more than 500 nm, that form through direct outward budding and shedding of the plasma membrane [22]. Their biogenesis involves cytoskeletal remodeling, calcium influx, and redistribution of membrane phospholipids, particularly the externalization of phosphatidylserine, mediated by enzymes such as scramblases and flippases [23,24,25,26,27]. Apoptotic bodies, which may exceed 1 µm in diameter, are generated during programmed cell death and contain fragmented cellular material, including organelles and nuclear components [28].

These differences in size and biogenetic origin are not merely structural but also have important functional consequences. Smaller, exosome-sized vesicles show enhanced tissue penetration and are readily internalized by non-phagocytic cells through endocytic pathways, including clathrin-mediated endocytosis and macropinocytosis [15,29]. In contrast, microvesicles and apoptotic bodies are more commonly taken up by professional phagocytes, such as macrophages and dendritic cells, thereby promoting antigen processing and presentation [2]. As a result, vesicle size and biogenesis influence biodistribution, cellular uptake, and immunological outcomes, which are critical considerations for therapeutic applications [3].

### 2.2. Bacterial Extracellular Vesicles (BEVs)

The biogenesis of BEVs is a complex process regulated by both genetic and environmental factors, but it is fundamentally constrained by bacterial cell envelope architecture and its dynamic remodeling [30]. In bacteria, EV production serves not only as a means of communication but also contributes to virulence and to the protection and transfer of nucleic acids [10].

Selective cargo sorting in BEVs begins early during vesicle formation, once membrane curvature is established [5,31]. Membrane-associated proteins are preferentially incorporated, often through sequence-dependent mechanisms, whereas essential cellular enzymes are excluded [31]. This process can enrich vesicles in virulence factors and antibiotic-degrading enzymes, thereby increasing their local concentration. BEV formation is also accompanied by changes in lipid composition, with vesicle membranes often enriched in specialized signaling lipids relative to the parent membrane [4,31]. In addition to proteins and lipids, nucleic acid packaging—particularly of RNA—is regulated by RNA-binding proteins that recognize structural motifs, facilitating the incorporation of these molecules into vesicles involved in horizontal gene transfer and host gene modulation [32,33]. Not all BEVs, however, arise through regulated cargo-sorting pathways. Alternative mechanisms, including explosive cell lysis, can generate vesicles containing cytoplasmic material, DNA, and membrane fragments released during bacterial disruption [34]. Overall, BEVs act as biologically active carriers of molecular information that support bacterial survival, adaptation, and host interaction [35].

The cargo composition of BEVs is closely linked to their biogenetic origin. Vesicles generated through regulated membrane budding may show selective enrichment of proteins, RNAs, signaling molecules, and virulence-associated factors that support bacterial survival and host interaction. By contrast, vesicles formed through explosive cell lysis arise from bacterial envelope disruption and may contain cytoplasmic constituents, DNA, and membrane fragments released during cell death. Accordingly, the degree of cargo selectivity can vary substantially among BEV subpopulations, reflecting differences in their biogenesis [30,34,35].

#### 2.2.1. Gram-Negative Bacterial Extracellular Vesicles

In Gram-negative bacteria, vesicle biogenesis primarily involves controlled outer membrane blebbing that occurs when local connections between the outer membrane and the underlying peptidoglycan layer are weakened. This process can be promoted by periplasmic protein accumulation or by curvature-inducing molecules embedded in the outer membrane. As the membrane protrudes and subsequently seals, a vesicle is released, capturing selected components of the periplasmic space [36].

EV composition and biological function are closely linked to vesicle origin and membrane architecture in Gram-negative species. Vesicles derived exclusively from the outer membrane are termed outer membrane vesicles (OMVs) [37]. These vesicles typically contain outer membrane components, including lipopolysaccharides (LPS), phospholipids, outer membrane proteins, and periplasmic proteins, including stress-associated factors such as heat-shock proteins, and may also contribute to the dissemination of antibiotic resistance determinants [38,39,40]. OMV formation is strongly influenced by the structural organization and lipid composition of the outer membrane [41]. Current models propose several mechanisms that may lead to vesicle formation. Current models propose several non-exclusive mechanisms of OMV biogenesis. One involves local changes in membrane properties caused by lipid or LPS remodeling, which generate curvature and promote vesicle budding [40,42]. A second mechanism suggests that increased periplasmic turgor drives outward bulging of the outer membrane, whereas a third proposes that reduced numbers of stabilizing links between the outer membrane and the peptidoglycan layer permit membrane blebbing and vesicle release [37].

In addition to classical OMVs, Gram-negative bacteria can produce vesicles that contain both outer and inner membrane components. These vesicles, often termed outer–inner membrane vesicles (OIMVs), are thought to arise through inward protrusion of the inner membrane into the periplasm before release, resulting in vesicles that also contain cytoplasmic material. Although OIMVs differ from mammalian exosomes in membrane architecture and biogenesis, both belong to the broader class of extracellular vesicles and share fundamental features, including a lipid bilayer boundary, cargo-transport capacity, and roles in intercellular communication. Gram-negative bacteria can also generate EVs through explosive cell lysis, a process in which the cell envelope ruptures and membrane fragments subsequently reassemble into spherical vesicles [43]. The resulting vesicles, often referred to as explosive outer membrane vesicles (EOMVs), can contain a broader range of cellular constituents, including DNA, cytoplasmic proteins, and inner membrane fragments, and may therefore facilitate horizontal gene transfer and biofilm formation [44]. Because these vesicles can encapsulate intracellular genetic material, this pathway is particularly relevant to the environmental dissemination of DNA [34].

#### 2.2.2. Gram-Positive Bacteria

In Gram-positive bacteria, vesicle biogenesis presents additional structural challenges because protrusions originating from the cytoplasmic membrane must traverse a thick and rigid peptidoglycan layer. This process is facilitated by specialized autolytic enzymes, known as autolysins, which generate localized perforations and channels within the cell wall [45]. Under the influence of internal turgor pressure, these openings permit vesicle passage without necessarily compromising cell viability. In this way, Gram-positive bacteria can release extracellular vesicles that support intercellular communication and may contribute to immune evasion [46].

### 2.3. Plant-Originated Extracellular Vesicles (PEVs)

Plant-derived EVs display considerable size heterogeneity, although reported distributions vary widely across studies. Many reports describe plant extracellular vesicles, often referred to as exosome-like nanovesicles, within size ranges comparable to mammalian small EVs, whereas other vesicle populations, including exocyst-positive organelle-derived (EXPO) vesicles, may extend into the 200–500 nm range [11,47]. As a result, plant EV preparations often appear more heterogeneous than mammalian EV isolates. Direct comparisons remain difficult, however, because reported size distributions are strongly influenced by plant species, tissue source, isolation method, and analytical approach. Thus, although a general trend toward broader size distributions is evident, no consensus has yet been reached on the characteristic size range of plant-derived EVs [48,49]. This diversity likely reflects multiple routes of plant EV biogenesis, the structural constraints imposed by the cell wall, and substantial variation among plant species and isolation protocols [50].

EVs have recently gained increasing attention as important mediators of intercellular communication in plants. Similar to their counterparts in mammalian systems, plant-derived EVs are membrane-bound nanoparticles that transport a diverse cargo, including proteins, lipids, small RNAs, and other signaling molecules [51]. These vesicles are secreted into the apoplast and contribute to multiple physiological processes, including immune responses [11]. Growing evidence indicates that plant EVs play a central role in plant–pathogen interactions by delivering defense-related molecules to invading microorganisms and facilitating communication between plant cells. This EV-mediated transfer of bioactive cargo is an important component of plant innate immunity and highlights the broader significance of EVs across kingdoms [51].

The mechanisms underlying EV formation in plants are still being elucidated, but current evidence indicates that multiple biogenetic pathways contribute to EV production [52]. One well-characterized route involves multivesicular bodies (MVBs), which generate intraluminal vesicles through inward budding of the endosomal membrane. Upon fusion of MVBs with the plasma membrane, these vesicles are released into the apoplast as exosome-like EVs [53]. In several plant species, including Arabidopsis and tobacco, the tetraspanin-family proteins TET8 and TET9 have been identified as markers of these vesicles [54,55]. TET8-positive EVs are enriched in defense-related proteins and small RNAs, including small interfering RNAs (siRNAs), and accumulate during pathogen infection, supporting their role in plant immune responses [56,57].

In addition to MVB-derived vesicles, plants also produce EVs through a distinct pathway involving EXPOs, which have been implicated in defense against fungal and bacterial infection [58]. These double-membrane structures are characterized by the exocyst subunit Exo70E2 and are thought to contribute to unconventional protein secretion [59]. EXPO-derived vesicles are proposed to fuse directly with the plasma membrane, thereby releasing their internal vesicles into the extracellular space. Although the functional differences between MVB-derived EVs and EXPO-derived vesicles remain under investigation, the existence of multiple secretion routes suggests that plants use diverse vesicle-trafficking mechanisms to regulate extracellular communication and deliver defense-related cargo [51,58].

## 3. Origin-Specific Immunology

**Figure 2:**
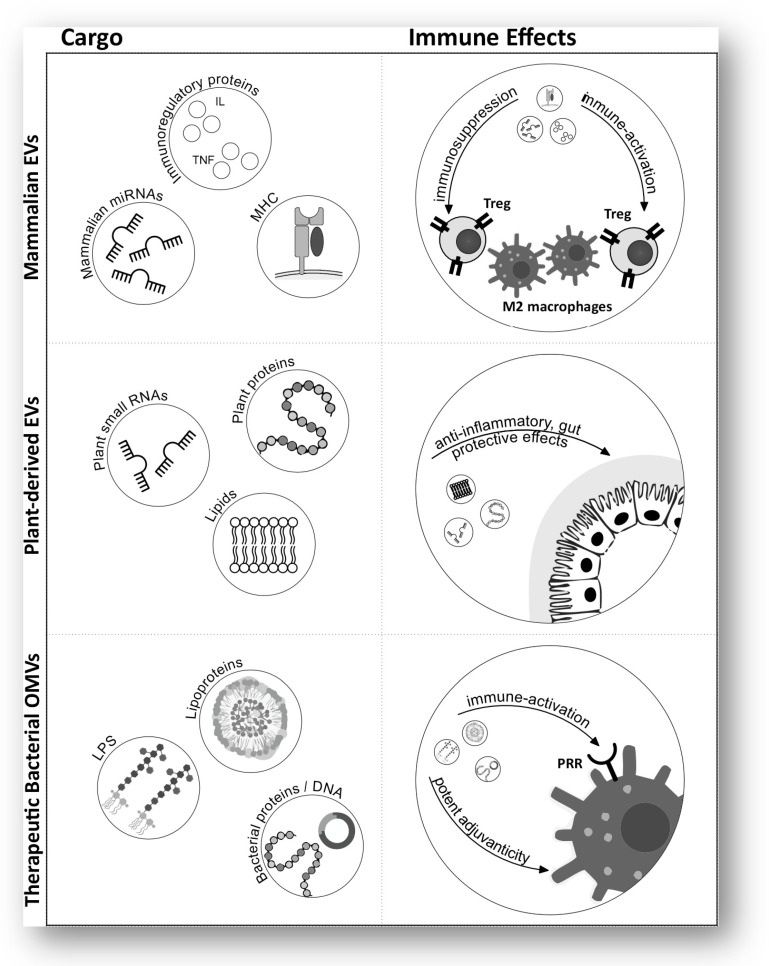
Immune effect summarization of the EVs across kingdoms.

EV origin is a key determinant of biogenesis and strongly influences biodistribution, cellular uptake, and immune outcomes. These features are shaped not only by vesicle size but also by the structural and physiological properties of the parent cell, which differ systematically across biological kingdoms [8]. In the context of immunology, EVs can be considered from two complementary perspectives: the physiological conditions and cellular processes that drive their release, and the immune responses they elicit in recipient host cells after uptake [9] (Figure 2).

### 3.1. Mammalian Extracellular Vesicles (MEVs) Immune Responses

In mammalian systems, the physiological roles of EVs and their immune-modulatory effects are closely interconnected. Rather than representing distinct processes, EV release and EV-mediated immune communication are integral components of the same regulatory networks that maintain immune homeostasis, coordinate inflammation, and support tissue repair. Accordingly, the following section discusses these functions together.

#### Physiological Roles of EVs in Mammalian Systems and Immune Modulation Mechanisms of the MEVs

In mammalian organisms, extracellular vesicles (EVs) function as highly specialized mediators of intercellular communication that coordinate tissue homeostasis, immune surveillance, cellular adaptation, and tissue repair. In multicellular systems, communication between spatially separated cell populations is essential, and EVs contribute to this process by transferring proteins, lipids, nucleic acids, metabolites, and other signaling molecules [17,60,61]. Through this cargo transfer, EVs enable cells to respond dynamically to developmental cues, environmental changes, tissue injury, and infection. As a result, mammalian EVs participate in both the initiation and resolution of immune responses, with their effects largely reflecting the physiological state of the parent cell [62].

Within the immune system, EVs contribute to antigen presentation, adaptive immune activation, immune regulation, and tissue regeneration. EVs derived from dendritic cells (DCs) and B cells carry major histocompatibility complex (MHC)–antigen complexes together with co-stimulatory molecules such as CD80 and CD86, thereby enabling either direct T-cell activation or the transfer of antigenic information to other antigen-presenting cells through a mechanism termed “cross-dressing” [63].

EVs derived from natural killer (NK) cells are enriched in cytotoxic effector molecules, including perforin, granzymes, and granulysin, which enable them to exert direct cytolytic activity against target cells [64]. Similarly, B-cell-derived EVs carry MHC class II molecules and functional B-cell receptors, thereby supporting antigen presentation and promoting antigen-specific immune responses [65].

Placenta-derived EVs are important for establishing and maintaining maternal–fetal immune tolerance during pregnancy, thereby supporting the specialized immunological environment required for fetal development [66].

EVs also play an important role in immune tolerance. Regulatory T cell (Treg)-derived EVs exert immunosuppressive effects by inhibiting effector T-cell proliferation and promoting the release of anti-inflammatory cytokines such as transforming growth factor-beta (TGF-β) and interleukin-10 (IL-10), thereby limiting inflammation and supporting immune regulation [66,67]. In addition, T cell-derived EVs can transport Fas ligand and APO2 ligand, both of which contribute to activation-induced cell death and help maintain immune homeostasis [68,69,70]. Beyond cellular origin, the immunological activity of EVs is strongly influenced by the nature and relative abundance of their molecular cargo. Anti-inflammatory mediators and regulatory microRNAs promote immune tolerance and support tissue repair [71,72].

By contrast, EV-associated mitochondrial DNA, extracellular DNA, heat shock proteins, and other danger-associated molecules can activate pattern-recognition receptors, including TLR9 and cGAS-STING signaling pathways, thereby promoting innate immune activation and inflammatory responses. Importantly, EV activity depends not only on the presence of these mediators but also on their relative abundance and molecular context. Consequently, vesicles with similar physical properties may elicit markedly different immune responses depending on cargo composition and enrichment [71]. These observations highlight the dynamic and context-dependent roles of immune cell-derived EVs in regulating immune function.

### 3.2. Bacterial EV Immune Responses

#### 3.2.1. Physiological Roles of BEVs Immune Response

In bacterial systems, EVs are integral to cellular physiology and are closely linked to envelope dynamics and stress adaptation [73]. Vesicle production is not merely constitutive but is modulated by environmental and cellular stressors, including oxidative stress, antibiotic exposure, nutrient limitation, and the accumulation of misfolded proteins in the periplasm [74,75]. Under these conditions, increased vesiculation functions as a protective response that helps maintain envelope integrity by removing damaged lipids, misfolded outer membrane proteins, and toxic compounds [76]. This process is often associated with envelope stress response pathways, such as RpoE-mediated signaling in Gram-negative bacteria, which regulate outer membrane protein folding, turnover, and membrane remodeling [76,77]. Although EV formation has been extensively studied in bacterial systems, the biological consequences of vesicle release extend well beyond microbial physiology. In multicellular organisms, bacterial EVs also act as important mediators of host interaction and immune modulation [78].

Beyond maintaining cellular homeostasis and mediating stress responses, bacterial EVs also facilitate communication among prokaryotic cells and contribute to microbial community dynamics [79]. By transporting signaling molecules, enzymes, and extracellular DNA, these vesicles support biofilm formation, matrix organization, and structural stability. Within biofilms, EVs may also carry quorum-sensing molecules that help coordinate collective bacterial behavior [79]. In addition, bacterial EVs are enriched in virulence-associated components, including LPS, invasins, adhesins, immunomodulatory compounds, and lytic enzymes [80]. Many bacteria also package antimicrobial factors into EVs, such as peptidoglycan hydrolases, which can lyse competing Gram-negative and Gram-positive bacteria. In this way, EV release enhances bacterial fitness, competitiveness, and survival in polymicrobial environments [43].

Bacterial EVs also facilitate horizontal gene transfer by encapsulating DNA, RNA, and plasmid fragments, thereby promoting the dissemination of antibiotic resistance genes and virulence factors [40,81]. Their composition and abundance depend strongly on the physiological state of the producing bacteria, with stress conditions often favoring the release of vesicles enriched in cytoplasmic material, particularly those generated through explosive cell lysis [4,34].

Overall, bacterial EV production represents a multifaceted strategy that integrates stress adaptation, envelope maintenance, and ecological competition. Rather than being passive byproducts, these vesicles function as dynamic extensions of the bacterial cell that promote survival in fluctuating and competitive environments.

#### 3.2.2. Immune Modulation Mechanisms of the BEVs in Host Cells

Bacterial EVs can strongly influence host immune responses by presenting pathogen-associated molecular patterns (PAMPs) that are recognized by innate immune pattern-recognition receptors (PRRs). These molecules are detected by host PRRs, particularly Toll-like receptors (TLRs), thereby activating innate immune signaling pathways. Because BEVs can arise through multiple biogenetic routes—including OMVs, OIMVs, and EOMVs—they contain a diverse repertoire of immunologically active molecules [82].

OMVs carry surface-exposed PAMPs such as LPS and lipoproteins. Their nanoscale size favors transport to draining lymph nodes and interaction with pattern-recognition receptors on innate immune cells, which underlie their strong adjuvant activity in vaccine settings [83]. The PAMPs are primarily recognized by TLR4, whereas bacterial lipoproteins activate TLR2-dependent signaling. Engagement of these receptors triggers downstream pathways, including NF-κB activation and pro-inflammatory cytokine production, thereby initiating innate immune responses and shaping subsequent adaptive immunity [82].

Growth phase and environmental stress can alter OMV size distributions, and some bacteria produce multilamellar or enlarged blebs under specific conditions, further illustrating the influence of parental physiology on vesicle size [44,84].

From an immunological and translational perspective, vesicle size influences both therapeutic utility and potential adverse effects. Smaller EVs tend to distribute more systemically and may modulate distal immune responses or cellular programs through delivery of microRNA (miRNAs) and protein cargo, which is advantageous for regenerative or tolerogenic approaches. Larger vesicles and OMVs, by contrast, are well suited to delivering PAMPs and antigenic proteins to antigen-presenting cells and lymph nodes, making them attractive platforms for vaccines and cancer immunotherapies while also raising important safety considerations, including reactogenicity and endotoxin burden [83,85,86,87].

Gram-positive bacterial EVs can carry toxins, including hemolysins, and may induce host cell death or modulate immune responses through the transfer of blood-coagulation-related factors [88,89]. They have also been reported to interact with and inhibit the complement system and to influence both T-cell-dependent adaptive immunity and B-cell-mediated humoral responses, including the lysis of IgM- and IgG-coated antigens [90,91,92].

### 3.3. Plant-Originated EV Immune Responses

#### 3.3.1. Physiological Roles of Plant-Originated EV Immune Responses

In plants, EVs play a central role in intercellular communication and defense-related signaling. These vesicles are actively involved in plant innate immunity, particularly in response to pathogen invasion [93].

Plant-derived EVs are increasingly recognized for their immunological functions, with exosome-like vesicles serving as carriers that deliver host small RNAs into fungal cells, thereby inhibiting fungal virulence mechanisms. The protein components of their cargo are also considered critical mediators of molecular recognition and signal transduction. These components play important roles in pathogen detection and in the initiation of immune signaling pathways [57,93].

A key mechanism underlying plant EV function is the delivery of siRNAs and miRNAs into invading pathogens, enabling cross-kingdom RNA interference. These RNAs are incorporated into RNA-induced silencing complexes (RISC) containing Argonaute proteins, particularly AGO1, and are processed by Dicer-like (DCL) enzymes prior to loading into EVs. Upon transfer into fungal cells, these RNAs target virulence-related genes, leading to gene silencing and reduced pathogenicity [57,94].

Plant EVs are also closely associated with pattern-triggered immunity (PTI). Membrane-bound pattern recognition receptors like Flagellin-Sensing 2 (FLS2) and Chitin Elicitor Receptor Kinase 1 (CERK1) identify PAMPs and initiate downstream signaling pathways, including MAP kinase routes and transcriptional changes [95]. EVs assist these processes by transporting defense-related proteins, such as pathogenesis-related (PR) proteins, proteases, and enzymes that modify the cell wall, into the apoplastic space, where they can directly engage with pathogens [96].

In addition to defense, plant EVs participate in physiological processes such as stress adaptation, tissue repair, and root development. Their cargo reflects environmental conditions and cellular status, suggesting that EV-mediated transport is integrated into broader plant stress-response networks, including salicylic acid- and jasmonic acid-dependent signaling pathways [97].

#### 3.3.2. Host Immune Modulation of PEVs

Plant-derived extracellular vesicles have garnered growing scholarly interest due to their capacity to interact with mammalian systems while exhibiting minimal intrinsic immunogenicity. Following oral administration, these vesicles remain stable in the gastrointestinal tract and engage intestinal epithelial and immune cells, thereby helping preserve gut barrier integrity and modulating innate immune responses [97,98,99].

Mechanistically, plant EVs can be internalized by mammalian cells via endocytic pathways, enabling delivery of bioactive cargo, including small RNAs, lipids, and proteins. These molecules may influence host gene expression and immune signaling pathways, although the precise mechanisms remain under investigation. Compared to bacterial EVs, plant EVs generally lack strong PAMPs, resulting in reduced activation of TLR signaling and lower pro-inflammatory responses. This property makes them attractive candidates for therapeutic delivery systems, particularly for nucleic acid-based therapies and anti-inflammatory applications [11,98,99,100].

However, the functional properties of plant EVs are highly dependent on their source, isolation method, and physicochemical characteristics. Studies using vesicles derived from edible plants such as ginger and grapefruit have demonstrated variability in cargo composition, uptake efficiency, and biological activity. This heterogeneity presents a major challenge for standardization, dosing, and reproducibility in clinical applications [101].

These properties support the maintenance of gut barrier integrity, the modulation of innate immune responses, and low systemic immunogenicity, collectively making plant EVs promising candidates for the oral delivery of small RNAs and pharmaceutical agents. However, heterogeneity in source material and isolation methods, including differences among vesicles derived from plants such as ginger and grapefruit, may strongly influence their functional properties and therapeutic efficacy [76,99,101].

The therapeutic applicability of extracellular vesicles closely aligns with the physiological functions they serve in their source organisms. Mammalian EVs are enriched in regulatory mediators that support tissue homeostasis, immune modulation, and intercellular repair, thereby underpinning their relevance in regenerative medicine and immunomodulatory interventions [18]. In contrast, bacterial outer membrane vesicles (OMVs) contain pathogen-associated molecular patterns that efficiently activate innate and adaptive immune pathways, making them promising platforms for vaccine development and adjuvant design despite important safety considerations [32,83]. Plant-derived EVs generally exhibit low intrinsic immunogenicity and can transport small RNAs and bioactive compounds across biological barriers, supporting their investigation as oral delivery systems [11]. Collectively, these observations indicate that EV origin shapes cargo composition, cellular interactions, immune signaling, and, ultimately, therapeutic suitability.

In summary, neither origin nor cargo alone can reliably predict immune outcomes without concurrent compositional and functional characterization. For the field to advance translationally, head-to-head comparisons using standardized isolation methods and orthogonal sizing techniques are needed to link size-defined subpopulations to specific immune readouts and potency metrics (Table 1).

## 4. Application of Extracellular Vesicles in Therapeutic Strategies

EVs have emerged as promising therapeutic tools due to their ability to transfer bioactive molecules and modulate immune responses. Importantly, the therapeutic potential of EVs is strongly dependent on their biological origin, as vesicles derived from plants, mammalian cells, and bacteria exhibit fundamentally different immunological properties. This diversity enables the rational selection of EV sources for specific clinical applications, ranging from anti-inflammatory therapies to vaccine development.

Despite the rapid expansion of EV research, several challenges continue to limit the translation of extracellular vesicles into routine therapeutic applications. Considerable progress has been achieved in the standardization of mammalian EV research through the MISEV guidelines, which provide recommendations for EV isolation, characterization, functional studies, and reporting practices. However, comparable frameworks remain underdeveloped for EVs derived from other biological kingdoms, particularly plant and bacterial systems, where differences in isolation procedures, nomenclature, and characterization methodologies continue to complicate direct comparisons between studies [1].

Furthermore, although numerous reports demonstrate promising immunomodulatory and therapeutic effects of mammalian-, bacterial-, and plant-derived EVs in experimental models, important questions regarding safety, biodistribution, dosage, long-term toxicity, and reproducibility remain unresolved and are still evolving [1,6]. This is particularly relevant for emerging therapeutic applications of plant-derived EVs and bacterial vesicles, where the relationship between cargo composition, immune activation, and potential adverse effects is not yet fully understood. While EV-based approaches already show substantial promise as diagnostic and prognostic tools, further mechanistic and translational studies are required before their therapeutic use can be broadly implemented in clinical practice. Future research should therefore focus on establishing standardized methodologies across all biological kingdoms, improving cargo characterization, clarifying dose–response relationships, and identifying the molecular mechanisms that govern EV-mediated biological effects. Such advances will be essential for translating the unique properties of EVs into safe and effective therapeutic strategies.

### 4.1. Therapeutic Strategies for MEVs

EVs derived from mammalian cells, human mesenchymal stem cells (MSC-EVs), and immune cell-derived EVs are widely investigated for their immunomodulatory and regenerative properties. These vesicles typically exert anti-inflammatory effects and contribute to immune regulation through multiple mechanisms.

MSC-derived EVs have been shown to promote macrophage polarization toward an anti-inflammatory M2 phenotype, inhibit dendritic cell maturation, and induce regulatory T cell (Treg) responses [105]. These effects are mediated by EV-associated cytokines, growth factors, and regulatory miRNAs, which collectively suppress excessive immune activation and support tissue repair. As a result, MSC-EVs are being explored in the treatment of autoimmune diseases, inflammatory disorders, and tissue injury [106].

Clinical translation of mammalian EVs is already underway. Multiple clinical trials are evaluating the safety and efficacy of MSC-derived EVs in regenerative medicine and inflammatory diseases (e.g., NCT05787288), and meta-analyses indicate a generally favorable safety profile [107]. In addition, immune cell-derived EVs are being investigated for their ability to enhance antigen presentation or modulate immune responses in cancer immunotherapy [108].

A particularly illustrative example of the relationship between physiological function and therapeutic application is provided by mitochondrial-containing extracellular vesicles (mitoEVs). Under physiological conditions, these vesicles contribute to intercellular metabolic communication by transferring mitochondrial components or intact mitochondria between cells, thereby supporting cellular adaptation and tissue homeostasis. This naturally occurring mechanism has attracted growing interest for regenerative medicine, where mitoEV-mediated mitochondrial transfer has been investigated as a potential strategy to restore bioenergetic function, reduce oxidative stress, and promote tissue repair following cellular injury [72,109,110]. Although the therapeutic potential of mitoEVs remains under active investigation, their development highlights how endogenous EV functions may inspire novel translational approaches.

### 4.2. Therapeutic Strategies for Bacterial-Derived EVs and Outer Membrane Vesicles

BEVs, particularly outer membrane vesicles (OMVs), represent a distinct class of therapeutic candidates with potent immunostimulatory properties. Unlike mammalian and plant EVs, which are more often explored for immune modulation or cargo delivery, OMVs are primarily investigated for their ability to activate innate immune responses [111].

As mentioned before, OMVs’ naturally immunostimulatory effect as intrinsic adjuvants makes them highly effective vaccine platforms by combining antigen delivery with immune activation [112]. To improve safety and broaden therapeutic applicability, several engineering strategies have been developed. These include detoxifying lipid A to reduce endotoxicity, genetically modifying bacterial strains to remove virulence factors, and optimizing antigen display on vesicle surfaces [113]. Such approaches have enabled the successful development of OMV-based vaccines, including licensed vaccines against *Neisseria meningitidis*, and continue to support research into their use against infectious diseases and in cancer immunotherapy [39,114].

However, the potent immunostimulatory nature of OMVs also raises safety considerations. Experimental studies have shown that OMVs can induce strong inflammatory responses, including activation of microglia and the NLRP3 inflammasome in the central nervous system. In mouse models, OMV exposure has been associated with neuroinflammation, disruption of blood–brain barrier integrity through downregulation of tight junction proteins such as zonula occludens-1 (ZO-1), occludin (OCLN), and claudin-5 (CLDN-5), and accumulation of β-amyloid, leading to cognitive impairment and tau phosphorylation [115,116]. These findings underscore the importance of carefully controlling OMV composition and dosing in therapeutic settings.

Despite their considerable therapeutic potential, several challenges continue to limit the clinical translation of bacterial EVs. The strong immunostimulatory properties that make OMVs attractive vaccine adjuvants may also provoke excessive inflammatory responses and reactogenicity [37,83,104]. Although genetic engineering and detergent-based detoxification strategies have reduced endotoxin-associated toxicity in several OMV-based platforms, maintaining an optimal balance between safety and immunogenicity remains a major challenge [85]. In addition, cargo composition can vary with bacterial strain, growth conditions, and vesicle isolation procedures, contributing to batch-to-batch variability and potentially affecting therapeutic consistency [31,76]. These limitations highlight the need for standardized production methods and comprehensive safety assessment in the future clinical development of bacterial EV-based therapeutics.

### 4.3. Therapeutic Strategies for PEVs

PEVs are increasingly recognized as low-immunogenic delivery systems with strong potential for oral and mucosal therapies. Their natural origin, stability in the gastrointestinal tract, and ability to carry small RNAs and bioactive compounds make them particularly attractive for therapeutic applications [114].

Plant-derived EVs have attracted considerable interest as delivery platforms due to their stability in the gastrointestinal tract, low intrinsic immunogenicity, and ability to transport diverse therapeutic cargoes [100,117,118]. A key mechanistic feature of PEVs is their involvement in cross-kingdom RNA interference. Plant EVs can deliver small RNAs into fungal or mammalian cells, where they regulate gene expression through RNA silencing pathways. This property has been proposed as a strategy for delivering pathogen-targeting RNAs in agriculture, but also highlights their broader potential as nucleic acid delivery systems [98].

Preclinical studies have demonstrated that PEVs can exert significant anti-inflammatory effects. For example, ginger-derived nanoparticles have been shown to reduce pro-inflammatory cytokine production, alleviate acute colitis, promote intestinal repair, and prevent the development of chronic colitis and colitis-associated cancer [114]. These effects are associated with the modulation of intestinal immune responses and the maintenance of epithelial barrier integrity. In addition to nucleic acids and conventional pharmaceutical agents, PEVs have also been proposed as carriers for naturally occurring bioactive compounds. Plant-derived polyphenols and bioactive organic acids have demonstrated anti-inflammatory, regenerative, and anticancer activities in experimental models, suggesting that EV-mediated delivery may further enhance their stability, bioavailability, and therapeutic potential [118].

In addition to anti-inflammatory applications, PEVs are being explored as carriers for drug delivery and nutraceutical formulations. Their low systemic immunogenicity and capacity to interact with gut epithelial and immune cells support their use in oral therapies and tumor-targeting strategies, although variability in source material and isolation methods remains a challenge for standardization [49].

Despite these encouraging findings, several factors continue to complicate the therapeutic development of PEV-based formulations. The biological composition of PEV preparations is strongly influenced by plant species, tissue source, growth conditions, extraction procedures, and isolation methodology, making direct comparisons between studies difficult [119]. Consequently, it remains unclear whether the observed therapeutic effects are primarily attributable to conserved vesicular mechanisms or to bioactive compounds naturally enriched in particular plant sources [120,121,122,123]. Furthermore, while oral administration is the most extensively investigated route of delivery, relatively limited information is available on dose–response relationships, biodistribution after repeated administration, and long-term safety. These questions become particularly important given reports that certain PEV populations may cross biological barriers, including the blood–brain barrier, potentially extending their systemic effects beyond the gastrointestinal tract [124]. These uncertainties are particularly relevant when considering systemic applications, where the immunological consequences of introducing plant-derived vesicles into mammalian tissues remain incompletely characterized. Therefore, although PEVs represent promising candidates for RNA delivery and mucosal therapies, additional mechanistic and translational studies are required before their therapeutic suitability can be fully assessed [11,12,124].

## 5. Conclusions

EVs have emerged as fundamental mediators of intercellular communication across biological kingdoms, with functions ranging from microbial ecology to complex immune regulation in multicellular organisms. Despite shared structural features, EVs derived from bacteria, plants, and mammalian cells exhibit distinct biological properties that reflect the physiological context and evolutionary pressures of their origin.

Taken together, the evidence reviewed here indicates that the immunological behavior and translational potential of EVs are determined chiefly by their cellular origin and molecular cargo.

BEVs, particularly OMVs, are enriched in PAMPs and act as potent activators of innate immunity through pathways that include TLR signaling and downstream NF-κB activation. In contrast, plant-derived EVs are characterized by low immunogenicity and the capacity to mediate cross-kingdom RNA interference, making them promising platforms for nucleic acid delivery and mucosal therapies. Mammalian EVs, especially those derived from stem cells and immune cells, predominantly exert regulatory effects by modulating cytokine signaling, antigen presentation, and immune cell differentiation.

These distinctions support the concept of “choosing the right EV” for a given therapeutic application. Bacterial EVs are well suited to vaccine development and other immunostimulatory strategies, whereas mammalian EVs are more appropriate for anti-inflammatory and regenerative interventions. Plant-derived EVs occupy an intermediate position as biocompatible delivery systems with minimal immunogenicity. Accordingly, the therapeutic promise of EVs lies not in a universal platform, but in the deliberate selection and engineering of vesicles with context-specific immunological properties.

Although the diagnostic value of EVs is increasingly supported by experimental and clinical evidence, their therapeutic application remains limited by unresolved challenges related to standardization, safety, dosing, and biological variability. Overcoming these barriers will require continued mechanistic investigation together with careful translational development.

Emerging engineering strategies are increasingly being explored to overcome some of the intrinsic limitations associated with naturally derived EVs. Surface modification approaches, targeted ligand display, cargo loading techniques, and hybrid vesicle platforms have been developed to improve cellular targeting, enhance therapeutic potency, and increase cargo consistency. In mammalian EV systems, engineering approaches have been used to enrich specific RNAs, proteins, or immunomodulatory molecules and to improve tissue-specific delivery. Similar strategies have been applied to bacterial OMVs to reduce endotoxin-associated toxicity while preserving immunostimulatory activity. In plant-derived EVs, engineered cargo loading and formulation approaches may help address variability associated with natural source materials. Although these technologies remain at various stages of development, they illustrate how EV engineering may bridge the gap between naturally occurring biological functions and the requirements of standardized therapeutic products.

Future research should integrate mechanistic insight with advances in EV engineering, including targeted cargo loading, surface modification, and scalable production methods. A deeper understanding of how EV origin, cargo, and physicochemical properties shape immune responses will be essential for the rational design of next-generation EV-based therapeutics. Further investigation of the relationships among EV biogenesis, cellular stress conditions, and functional outcomes will be necessary to enable more precise control over vesicle composition and activity.

In conclusion, EVs represent a versatile and biologically grounded platform for immunomodulation and drug delivery. By leveraging the inherent diversity of EVs across biological kingdoms, it may be possible to develop tailored therapeutic strategies that harness their distinct immunological functions. This cross-kingdom perspective provides a useful conceptual framework for advancing EV research from descriptive biology toward more precise translational applications.

## Figures and Tables

**Table 1:** Summary of cross-kingdom extracellular vesicles.

EV Source/Subtype	Representative Cargo	Typical Recipient Cells	Uptake/Interaction Mechanism	Major Immune Receptors/Pathways	Dominant Immune Effect	Therapeutic Applications	Evidence Level	Key Translational Challenges	References
Mammalian EVs (general)	miRNAs, cytokines, growth factors, lipids	Immune cells, epithelial cells, stromal cells	Endocytosis, membrane fusion, receptor-mediated uptake	NF-κB modulation, antigen presentation pathways	Immune regulation, tissue homeostasis	Drug delivery, immunomodulation	Clinical trials/Preclinical	Heterogeneity, manufacturing	[68,102]
EVs derived from immune cells (DC, B-cell, T-cell, NK-cell)	MHC-antigen complexes, CD80/CD86, perforin, granzymes, FasL	T cells, APCs, NK cells	Receptor-mediated interactions, antigen transfer	Antigen presentation pathways, T-cell activation	Immune activation or tolerance, depending on source	Vaccines, immunotherapy	Mostly preclinical	Source-specific variability	[2,9,63,65,103]
EVs derived from mesenchymal stem cells (MSCs)	TGF-β, IL-10, regulatory miRNAs, growth factors	Macrophages, T cells, injured tissues	Endocytosis, paracrine signaling	TGF-β signaling, NF-κB suppression	Anti-inflammatory, regenerative	Regenerative medicine, inflammatory diseases	Clinical trials/Preclinical	Standardization, donor variability, dosage	[104]
Plant-derived EVs (PEVs)	Small RNAs, metabolites, polyphenols, lipids	Gut epithelial cells, macrophages, intestinal immune cells	Intestinal uptake, endocytosis, cross-kingdom RNA transfer	Incompletely characterized; RNA-mediated regulation	Low inflammatory profile, barrier modulation	Oral delivery, RNA delivery, nutraceuticals	Predominantly preclinical	Source variability, isolation variability, limited clinical evidence	[48,54,84]
Gram-negative OMVs	LPS, outer membrane proteins, bacterial DNA/RNA	APCs, macrophages, dendritic cells	PRR recognition, endocytosis	TLR4, TLR2, TLR9, cGAS-STING	Strong immune activation	Vaccines, adjuvants	Approved vaccines/Preclinical	Reactogenicity, endotoxin toxicity	[44,82]
Gram-positive EVs	Lipoproteins, peptidoglycan fragments, proteins, nucleic acids	APCs, epithelial cells	Endocytosis, PRR engagement	TLR2, NOD-like receptors	Immune activation	Vaccines, antimicrobial applications	Preclinical	Limited characterization	[20,91]

## Data Availability

In this review, no new data were created.

## Abbreviations

The following abbreviations are used in this manuscript:

ALIX	Programmed cell death 6-interacting protein
BEV	Bacterial extracellular vesicle
CERK1	Chitin elicitor receptor kinase 1
DC	Dendritic cell
DCL	Dicer-like
EOMV	Explosive outer membrane vesicle
ESCRT	Endosomal sorting complexes required for transport
EV	Extracellular vesicle
EXPO	Exocyst-positive organelle
FLS2	Flagellin-sensing 2
IL-10	Interleukin-10
LPS	Lipopolysaccharide
MEV	Mammalian extracellular vesicle
miRNAs	MicroRNA
MSC-EV	Mesenchymal stem cell-extracellular vesicle
MVB	Multivesicular body
NF-κB	Nuclear factor kappa-light-chain-enhancer of activated B cells
OIMV	Outer-inner membrane vesicle
OMV	Outer membrane vesicle
PAMP	Pathogen-associated molecular pattern
PEV	Plant-originated extracellular vesicle
PR	Pathogenesis-related
PRR	PAMP recognition receptor
PTI	Pattern-triggered immunity
RISC	RNA-induced silencing
siRNAs	Small interfering RNA
TGF-β	Transforming growth factor-beta
TLR	Toll-like receptor
Treg	Regulatory T cell
TSG101	Tumor Susceptibility Gene 101
